# Protective Response Mechanisms to Heat Stress in Interaction with High [CO_2_] Conditions in *Coffea* spp.

**DOI:** 10.3389/fpls.2016.00947

**Published:** 2016-06-29

**Authors:** Madlles Q. Martins, Weverton P. Rodrigues, Ana S. Fortunato, António E. Leitão, Ana P. Rodrigues, Isabel P. Pais, Lima D. Martins, Maria J. Silva, Fernando H. Reboredo, Fábio L. Partelli, Eliemar Campostrini, Marcelo A. Tomaz, Paula Scotti-Campos, Ana I. Ribeiro-Barros, Fernando J. C. Lidon, Fábio M. DaMatta, José C. Ramalho

**Affiliations:** ^1^Grupo Interações Planta-Ambiente and Biodiversidade (PlantStress&Biodiversity), Departamento Recursos Naturais, Ambiente e Território (DRAT), Linking Landscape, Environment, Agriculture and Food (LEAF), and Forest Research Center (CEF), Instituto Superior de Agronomia, Universidade de LisboaOeiras, Portugal; ^2^Departamento Ciências Agrárias e Biológicas, Centro Universitário Norte do Espírito Santo, Universidade Federal Espírito SantoSão Mateus, Brazil; ^3^Setor Fisiologia Vegetal, Centro de Ciências e Tecnologias Agropecuárias, Universidade Estadual do Norte FluminenseRio de Janeiro, Brazil; ^4^GeoBioTec, Faculdade Ciências Tecnologia, Universidade NOVA de LisboaCaparica, Portugal; ^5^Unidade de Investigação em Biotecnologia e Recursos Genéticos, Instituto Nacional de Investigação Agrária e VeterináriaOeiras, Portugal; ^6^Departamento Produção Vegetal, Centro de Ciências Agrárias, Universidade Federal do Espírito SantoAlegre, Brazil; ^7^Departamento Biologia Vegetal, Universidade Federal de ViçosaViçosa, Brazil

**Keywords:** acclimation, antioxidants, coffee, chloroplast, climate change, enhanced [CO_2_], global warming, heat

## Abstract

Modeling studies have predicted that coffee crop will be endangered by future global warming, but recent reports highlighted that high [CO_2_] can mitigate heat impacts on coffee. This work aimed at identifying heat protective mechanisms promoted by CO_2_ in *Coffea arabica* (cv. Icatu and IPR108) and *Coffea canephora* cv. Conilon CL153. Plants were grown at 25/20°C (day/night), under 380 or 700 μL CO_2_ L^−1^, and then gradually submitted to 31/25, 37/30, and 42/34°C. Relevant heat tolerance up to 37/30°C for both [CO_2_] and all coffee genotypes was observed, likely supported by the maintenance or increase of the pools of several protective molecules (neoxanthin, lutein, carotenes, α-tocopherol, HSP70, raffinose), activities of antioxidant enzymes, such as superoxide dismutase (SOD), ascorbate peroxidase (APX), glutathione reductase (GR), catalase (CAT), and the upregulated expression of some genes (*ELIP, Chaperonin 20*). However, at 42/34°C a tolerance threshold was reached, mostly in the 380-plants and Icatu. Adjustments in raffinose, lutein, β-carotene, α-tocopherol and HSP70 pools, and the upregulated expression of genes related to protective (*ELIPS, HSP70, Chape 20*, and *60*) and antioxidant (*CAT, CuSOD2, APX Cyt, APX Chl*) proteins were largely driven by temperature. However, enhanced [CO_2_] maintained higher activities of GR (Icatu) and CAT (Icatu and IPR108), kept (or even increased) the Cu,Zn-SOD, APX, and CAT activities, and promoted a greater upregulation of those enzyme genes, as well as those related to HSP70, ELIPs, Chaperonins in CL153, and Icatu. These changes likely favored the maintenance of reactive oxygen species (ROS) at controlled levels and contributed to mitigate of photosystem II photoinhibition at the highest temperature. Overall, our results highlighted the important role of enhanced [CO_2_] on the coffee crop acclimation and sustainability under predicted future global warming scenarios.

## Introduction

Anthropogenic actions, mostly related to rising use of fossil fuels and land-use changes, have strongly contributed to the increase of atmospheric [CO_2_] from ca. 280 in the pre-industrial period to the actual 400 μL CO_2_ L^−1^. According to recent projections, depending on the greenhouse gas emission scenarios air [CO_2_] could rise to between 421 and 936 μL CO_2_ L^−1^, (or between 475 and 1313 μL CO_2_ L^−1^, considering CO_2_-equivalent concentrations that include the predicted concentrations of CH_4_ and N_2_O) by 2100. These will be accompanied by a global surface warming between 0.3 and 1.7°C (best scenario) and 2.6–4.8°C (worst scenario, without additional mitigation efforts), relative to 1986–2005 (IPCC, 2013, 2014).

This [CO_2_] boost can affect fundamental plant processes such as photosynthesis and respiration (Woodward, 2002; Ainsworth and Rogers, 2007; Kirschbaum, 2011), ultimately altering plant growth, yield, and crop quality (Luo et al., 1999; DaMatta et al., 2010). The actual atmospheric [CO_2_] is below the optimal for photosynthesis of C_3_ crops. Therefore, enhanced [CO_2_] is expected to increase net photosynthesis near or often above 50%, with trees showing the largest rise (Ainsworth and Rogers, 2007). However, projected global warming, concurrent to [CO_2_] increase may compromise the beneficial effects of C fertilization, and affect flower viability, fruit development, and yield (Prasad et al., 2003; Camargo, 2010), therefore constituting a major threat for many important agricultural crops. Indeed, heat modifies the use of solar energy, alters both the gas diffusion in the leaf mesophyll (Lambers et al., 2008) and photosynthetic pigment composition and content (Haldimann and Feller, 2004; Hasanuzzaman et al., 2013). Elevated temperatures also affect the water relations and evaporative demand, the fluidity and stability of membrane systems, as well as hormones and primary and secondary metabolites (Wahid et al., 2007). Collectively, these alterations could potentially disrupt cellular homeostasis and impair photosynthesis and other major physiological processes (Long et al., 2006; Suzuki and Mittler, 2006; Kirschbaum, 2011). In any case, elevated [CO_2_] can modify plant responses to environmental variables (Boisvenue and Running, 2006). Therefore, to properly predict plant responses to rising temperatures and atmospheric [CO_2_], studies must be undertaken considering both variables on a long-term basis, given that their interaction can either exacerbate or cancel their single independent effects on leaf physiology (Way et al., 2015).

The harmful effects of heat stress is frequently linked to the enhanced accumulation of reactive oxygen species (ROS) (Suzuki and Mittler, 2006; Hasanuzzaman et al., 2013). The upregulation of mechanisms controlling the production/scavenging of highly reactive molecules is determinant to cell homeostasis and to plant survival to environmental stresses. Chloroplast (and cell) oxidative stress usually arises under conditions that reduce energy use by photochemical processes without a significant reduction in energy capture. This imbalance promotes the over-production of molecules in the excited state, both of Chl and O_2_ [e.g., triplet state of chl (^3^Chl^*^), ^1^chl, singlet oxygen (^1^O_2_)]. Furthermore, O_2_ reduction in photosystems (particularly in PSI) may occur, producing ${\text{O}}_{2}^{\cdot –}$ and, thereafter, H_2_O_2_ and OH^·^, all of which can cause lipid peroxidation, bleaching of pigments (e.g., in P_680_), protein oxidation (e.g., D1), enzyme inactivation, and DNA degradation (Asada, 1994; Foyer, 2002; Logan, 2005). The control of such highly reactive molecules can be achieved indirectly, through the increase of energy dissipation mechanisms (e.g., photorespiration, pseudocyclic electron transport, and the synthesis of photoprotective pigments; Logan, 2005; Smirnoff, 2005; Batista-Santos et al., 2011), or directly, acting on their production/scavenging. The latter includes the complementary overexpression of enzymes (e.g., SOD, APX, GR), as well as the action of hydrophilic (ascorbate and glutathione) and lipophilic (e.g., zeaxanthin, β-carotene, and α-tocopherol) antioxidants (Foyer, 2002; Logan, 2005; Munné-Bosch, 2005; Smirnoff, 2005).

Coffee, an evergreen tropical tree species, is one of the most heavily globally traded commodities. The world coffee production is based on *Coffea arabica* L. and *C. canephora* Pierre ex A. Froehner, which produce ~2/3 and 1/3 of crop yield, respectively. The optimal annual average temperature is one of the well-known differences between *C. arabica* (18–23°C) and *C. canephora* (22–26°C) (DaMatta and Ramalho, 2006). Coffee crop chain of value generates an income of approximately US$ 170,000 million and involves ca. 100 million people worldwide, having a strong social and economic impact on many tropical countries (Bunn et al., 2015). Noticeably, the coffee production is mostly based on small holders that are currently facing growing challenges from climate changes; under these circumstances, both the coffee yields and beverage quality can be profoundly impacted, thus potentially affecting not only producers but also the coffee industry and consumers as a whole. In fact, over the last decade, several modeling studies anticipated remarkable climate impacts on the coffee crop (especially *C. arabica*), with large worldwide losses on suitable areas and productivity, although coffee production is believed to have already been affected by climate changes due to the occurrence of severe droughts and high temperatures (Bunn et al., 2015; Craparo et al., 2015; van der Vossen et al., 2015). However, neither the positive effects of elevated [CO_2_] on coffee photosynthesis (Ramalho et al., 2013b; DaMatta et al., 2016), leaf mineral balance (Martins et al., 2014), and bean yield (Ghini et al., 2015), nor the role of CO_2_ as a key player in coffee heat tolerance (Rodrigues et al., 2016), have been considered in those modeling studies, probably because this information has become available only very recently.

We previously demonstrated that both *C. arabica* and *C. canephora* species are remarkably heat tolerant up to 37/30°C, but at 42/34°C a threshold for irreversible non-stomatal deleterious effects on photosynthesis is reached, with greater impairments on *C. arabica*, particularly under normal air [CO_2_] (Rodrigues et al., 2016). Photosystems and thylakoid electron transport were shown to be quite heat tolerant, contrasting to the enzymes related to energy metabolism, including RuBisCO, which were the most sensitive components. We also demonstrated that elevated [CO_2_] does not provoke photosynthetic downregulation and remarkably mitigates the impact of temperature on both species, particularly at 42/34°C, modifying the response to supra-optimal temperatures. Here we complement our previous studies with the central objective of exploring potential protective mechanisms against heat stress, and how these mechanisms are affected by the elevated [CO_2_] in coffee. To reach these goals, we firstly assessed the occurrence of PSII photoinhibition (as a marker for photodamage), followed by an in-depth characterization of protective mechanisms, including dynamics of photosynthetic pigments, enzymatic and non-enzymatic antioxidant systems, quantification of raffinose family oligosaccharides (RFOs), and heat-shock protein pools, in addition to assessing the expression of selected genes with potential roles in heat acclimation.

## Materials and methods

### Plant material and experimental conditions

The plant materials were collected from the same experiments described in Rodrigues et al. (2016), therefore, using the same experimental conditions and treatments. In brief, three widely cropped coffee genotypes from the two main producing species, *C. arabica* L. (cvs. Icatu and IPR108) and *C. canephora* Pierre ex A. Froehner (cv. Conilon Clone 153—CL153), were evaluated. Plants, ca. 1.5 years of age, were transferred into two walk-in growth chambers (EHHF 10000, ARALAB, Portugal) differing in air [CO_2_] supply: 380 μL CO_2_ L^−1^ (380-plants) or 700 μL CO_2_ L^−1^ (700-plants). Both groups of plants were then grown for 10 months in 28 L pots in a substrate consisting of a mixture of soil, peat, and sand (3:1:3, v/v/v), as optimized for coffee plants (Ramalho et al., 2013a), and fertilized as previously described (Ramalho et al., 2013b). Environmental conditions of temperature (25/20°C, day/night), irradiance (ca. 700–800 μmol m^−2^ s^−1^), RH (75%), and photoperiod (12 h) were provided to the plants, and permanently monitored along the whole period. The temperature was then gradually increased from 25/20°C up to 42/34°C in both growth chambers at a rate of 0.5°C day^−1^, with 7 days of stabilization at 31/25, 37/30, and 42/34°C for evaluations.

All determinations were performed on newly matured leaves from the upper (illuminated) part of each plant. For biochemical evaluations, leaf material was collected after ca. 2 h of illumination from 6 to 8 plants of each genotype and used immediately or flash frozen in liquid N_2_ and stored at −80°C until analysis. Each biological replicate is a pool of leaves of each plant. Along the experiment the plants were maintained without restrictions in water, nutrients, and root development, the latter evaluated by visual examination at the end of the experiment after removing the plants from their pots (Rodrigues et al., 2016).

### PSII photoinhibition status

The PSII photoinhibition indexes were calculated according to Werner et al. (2002), and included: (A) chronic photoinhibition (PI_Chr_), representing the percent reduction in F_v_/F_m_ at each temperature relative to the maximal F_v_/F_m_ obtained during the entire experiment; (B) dynamic photoinhibition (PI_Dyn_), representing the decline in F_v_/F_m_ that is fully reversible overnight, being measured as the percent reduction in midday ${\text{F}}_{\text{v}}^{'}$/${\text{F}}_{\text{m}}^{'}$ relative to F_v_/F_m_ at each temperature, relative to the maximal F_v_/F_m_ from the entire experiment; (C) total photoinhibition (PI_Total_ = PI_Chr_ + PI_Dyn_). The F_v_/F_m_ and ${\text{F}}_{\text{v}}^{'}$/${\text{F}}_{\text{m}}^{'}$ represented the maximal photochemical efficiency of PSII and the actual PSII efficiency of energy conversion under light exposure, respectively. F_v_/F_m_ and ${\text{F}}_{\text{v}}^{'}$/${\text{F}}_{\text{m}}^{'}$ were obtained under dark adapted or photosynthetic steady-state conditions, respectively, exactly as detailed in Rodrigues et al. (2016).

### Photosynthetic pigment characterization

Carotenoids (Car) were assessed from four frozen leaf discs (each 0.5 cm^2^). Sample processing and subsequent reverse-phase HPLC were carried out as optimized for coffee (Ramalho et al., 1997) with minor adjustments, using an end-capped (C_18_) 5-μm Spherisorb ODS-2 column (250 × 4.6 mm). Detection was performed at 440 nm in an HPLC system (Beckman, System Gold, Tulsa, USA) coupled to a diode-array (Model 168; Beckman) detector, and identification and quantification were performed using individual sugar standards. The de-epoxidation state, involving xanthophyll cycle components, was calculated as DEPS = (Zeaxanthin (Z) + 0.5 Antheraxanthin (A)) / (Violaxanthin(V) + A + Z).

Chlorophylls (Chls) from the same samples were extracted in 80% acetone, and quantified spectrophotometrically according to Lichtenthaler (1987).

### Maximal activities of antioxidant enzymes

Unless otherwise indicated, enzyme activities were determined in chloroplast extracts (obtained using 3–4 g FW of leaf tissue), as described previously (Ramalho et al., 1998). Superoxide dismutase (Cu,Zn-SOD, EC 1.15.1.1) was spectrophotometrically assessed at 550 nm. Ascorbate peroxidase (APX, EC 1.11.1.11) was assessed through ascorbate consumption (at 290 nm, 120 s, 25°C) using an extinction coefficient of 2.8 mM^−1^ cm^−1^ for calculations. Glutathione reductase (GR, EC 1.6.4.2) was assessed through the NADPH oxidation (at 340 nm, 120 s, 25°C). Catalase (CAT, EC 1.11.1.6) was assessed in whole-leaf extracts (prepared using 200 mg FW of leaf tissue), as described in Fortunato et al. (2010). Enzyme activity was evaluated through the rate of H_2_O_2_ consumption (240 nm, 120 s, 25°C), and a freshly made H_2_O_2_ standard curve (0–1 M) was used for quantification.

### Quantification of non-enzymatic antioxidants

Ascorbate (ASC) and α-tocopherol (TOC) determinations were performed on 100 and 200 mg FW of leaf tissue, respectively, based on HPLC methods, as described in detail in Fortunato et al. (2010).

### HSP70 quantification

For the heat shock protein 70 (HSP70) assays, 100 mg FW of frozen leaf tissues per plant were homogenized in 1 mL 200 mM Tris-HCl (pH 8.0), containing 20 mM β-mercaptoethanol, 2 mM dithiothreitol (DTT), 2% triton X-100, 4% (v/v) “Complete-protease inhibitor cocktail” with EDTA, 10% polyvinylpolypyrrolidone, and 10% glycerol. The homogenate was then centrifuged (10,000 g, 20 min, 4°C) and the supernatant was used for HSP70 quantification through Enzyme Linked Immunosorbent Assay (ELISA), using Flat-bottomed micro-ELISA plates (Costar, Corning, NY, USA) as described in Njemini et al. (2003) with minor modifications. Plates were covered with the primary antibody (100 μL; 5 μg mL^−1^) diluted in 100 mM carbonate buffer (pH 9.6). After incubating the plates at 37°C during 90 min, the coated plates were washed four times with phosphate-buffered saline (PBS) containing 0.1% Tween 20 (PBS/T) and non-specific binding sites blocked by incubation with 300 μL of PBS/T containing 0.1% BSA (PBS/T/BSA) for 60 min at 37°C. After washing, 50 μL of the standard and samples were added and the plates incubated for 90 min at 37°C. Plates were then washed four times and 100 μL of mouse monoclonal anti-HSP70 (1/400) diluted in PBS/T were added. After 60 min on a shaker at 37°C, plates were washed four times and incubated with 100 μL of the diluted (1/10,000 in PBS/T/BSA) secondary antibody (anti-mouse IgG, peroxidase, Sigma-Aldrich, USA) for 60 min at 37°C. Thereafter, plates were washed and 100 μL of ABTS (2,2′-Azino-bis-3-ethylbenzothiazoline-6-sulfonic acid) substrate were added. After 45 min at 37°C, the reaction was stopped with 50 μL of 1 N H_2_SO_4_ and the absorbance was determined at 405 nm using a microplate reader. HSP70 concentrations were detected by comparing sample absorbance with the absorbance of a reference purified HSP70 protein.

Total soluble protein contents of the enzyme extracts followed Bradford (1976), using bovine serum albumin (BSA) as a standard.

### Raffinose family oligosaccharides quantification

RFOs evaluation was performed in samples of 150 mg of powdered frozen material, following the HPLC method for soluble sugars described in Ramalho et al. (2013b), with some modifications. To overcome the presence of non-pure peaks the separation of sugars was performed using a Sugar-Pak 1 column (300 × 6.5 mm, Waters) at 90°C, with H_2_O as the eluent (containing 50 mg EDTA-Ca L^−1^ H_2_O) and a flow rate of 0.5 mL min^−1^. Another 20 μL aliquot of each sample was injected through a DionexCarboPac PA1 analytical column (4 × 250 mm, Thermo Scientific, USA) coupled to a DionexCarboPac PA1 Guard (4 × 50 mm) at 20°C. Ultrapure water and 300 mM NaOH were used as eluents (water from 0 to 50 min; NaOH from 50 to 65 min; and water from 65 to 80 min for re-equilibration) at a 1 mL min^−1^ flow rate. A refractive index detector (Model 2414, Waters, USA) was used for sugar detection. Sugars were quantified using specific standard curves.

### Expression studies of selected genes

Total RNA was isolated and quantified as described in Goulao et al. (2012). One microgram of DNA-free total RNA was used to synthesize first-strand cDNAs using oligo-(dT)18 primers and the SuperScript II first-strand synthesis system (Invitrogen, USA).

Genes related to the antioxidative system and other protection proteins were selected for the expression studies (Tables 1, **6**), with all procedures performed as described in Goulao et al. (2012). Primers were designed using the *C. canephora* sequences (http://www.coffee-genome.org/coffeacanephora) with Primer3 software. Primer length was set to 19–20 bp, with a GC content between 45 and 60% and a melting temperature (Tm value) between 62 and 65°C. Amplicon length ranges were set to be between 80 and 150 bp. The probability of formation of hairpin structures and primer dimerization was subsequently checked using the Oligo Calculator algorithm. To determine the specificity of the primer pairs used in this study, melting/dissociation curve analysis was performed following the RT-qPCR experiment. A single peak in the obtained melting curve confirmed the specificity of the amplicon, and no signal was detected in the negative controls for all of the tested primers. All qRT-PCR reactions and relative gene expression were calculated after normalization with the reference genes ubiquitin-conjugating enzyme E2 (UbQ2) and eukaryotic initiation factor 4α(eLF-4).

**Table 1:** **Selected genes used for real-time qPCR studies, which are related to the oxidative stress control and/or repair mechanisms, homologies, primer sequences, access number on NCBI GenBank and amplicon size (bp)**.

**Gene symbol**	**Primer sequence (5′–3′)**	**Gene description**	**NCBI GenBank acess number**	**Amplicon size (bp)**
UBQ^*^	F: AACATTGAGGGTGGTTCTGTTC	Ubiquitin	AF297089	200
	R: CTCCAAGTGCACCTCAAACTC			
*HSP70*	F: GGGAAGCAATTGACACCAAG	Stromal 70 kDa heat shock-related protein, chloroplastic	GSCOCT00018441001;Name = Cc10_g11150	150
	R: AGCCACCAGATACTGCATCC			
*ELIP*	F:GCCATGATAGGGTTTGTTGC	Chloroplast early light-induced protein	GSCOCT00026140001;Name = Cc03_g04300	101
	R: GTCCCAATGAACCATTGCAG			
*Chape 20*	F: GTTAAAGCTGCCGCTGTTG	Chloroplast 20 kDa chaperonin	GSCOCT00041526001;Name = Cc06_g12530	150
	R: CTCACCTCCTTGAGGTTTCG			
*Chape 60*	F: GGATAGTGAAGCCCTTGC	Mitochondria chaperonin CPN60	GSCOCT00026540001;Name = Cc03_g07040	80
	R: CCCAGGAGCTTTTATTGCAC			
*CAT*	F: CTACTTCCCCTCGCGGTAT	Catalase isozyme 1	GSCOCT00036969001;Name = Cc07_g11710	150
	R: CTGTCTGGTGCAAATGAACG			
*CuSOD1*	F: CCCTTGGAGACACAACGAAT	Superoxide dismutase [Cu-Zn]	GSCOCT00012962001;Name = Cc02_g32280	141
	R: GGCAGTACCATCTTGACCA			
*CuSOD2*	F: GGGGCTCTATCCAATTCCTC	Superoxide dismutase [Cu-Zn]	GSCOCT00040610001;Name = Cc06_g23170	150
	R: GGTTAAAATGAGGCCCAGTG			
*FeSOD*	F: TGTCAACCCTCTTGTGTGGA	Chloroplast superoxide dismutase [Fe]	GSCOCT00031536001;Name = Cc10_g09500	141
	R: ATTGCCGCATTCCAAGATAC			
*APX Cyt*	F: TCTGGATTTGAGGGACCTTG	Cytosol ascorbate peroxidase	GSCOCT00023633001;Name = Cc06_g03490	108
	R: GTCAGATGGAAGCCGGATAA			
*APX Chl*	F: CACCTGCTGCTCATTTACG	Chloroplast ascorbate peroxidase	GSCOCT00031893001;Name = Cc10_g12080	100
	R: GACCTTCCCAATGTGTGTG			
*Toc Mt*	F: GCAGATGGGTCATTCGATTT	Chloroplast probable tocopherol O-methyltransferase	GSCOCT00040496001;Name = Cc06_g18700	146
	R: GGCGAAAGATCCCTATGAC			
*Toc Cy*	F: CCTAACTTTTGGGGAAGC	Chloroplast tocopherol cyclase	GSCOCT00017269001;Name = Cc08_g09240	150
	R: GATGCCAAAGGGGAGTAAC			

* *Used to check for DNA contamination in RNA samples and positive control for cDNA synthesis*.

### Statistical analysis

Data were analyzed using two-way ANOVAs (*P* < 0.05) to evaluate the differences between the two growth atmospheric [CO_2_] and among the several temperature treatments, as well as their interaction, followed by a Tukey's test for mean comparisons, except when otherwise stated. Each ANOVA was performed independently for each of the studied genotypes. Overall, the [CO_2_] × temperature interaction for most parameters was significant. To the sake of simplicity we do not considered also the comparison between genotypes within each [CO_2_] and temperature.

The relative expression ratio of each target gene was computed based on its real-time PCR efficiency and the crossing point (CP) difference of a target sample vs. a control (25/20°C, 380 μL CO_2_ L^−1^ air) within each genotype. Data analysis was performed using the Relative Expression Software Tool (REST 2009), available at http://www.genequantification.de/rest-2009.html. A 95% confidence level was adopted for all tests.

## Results

### PSII photoinhibition

**Figure 1:**
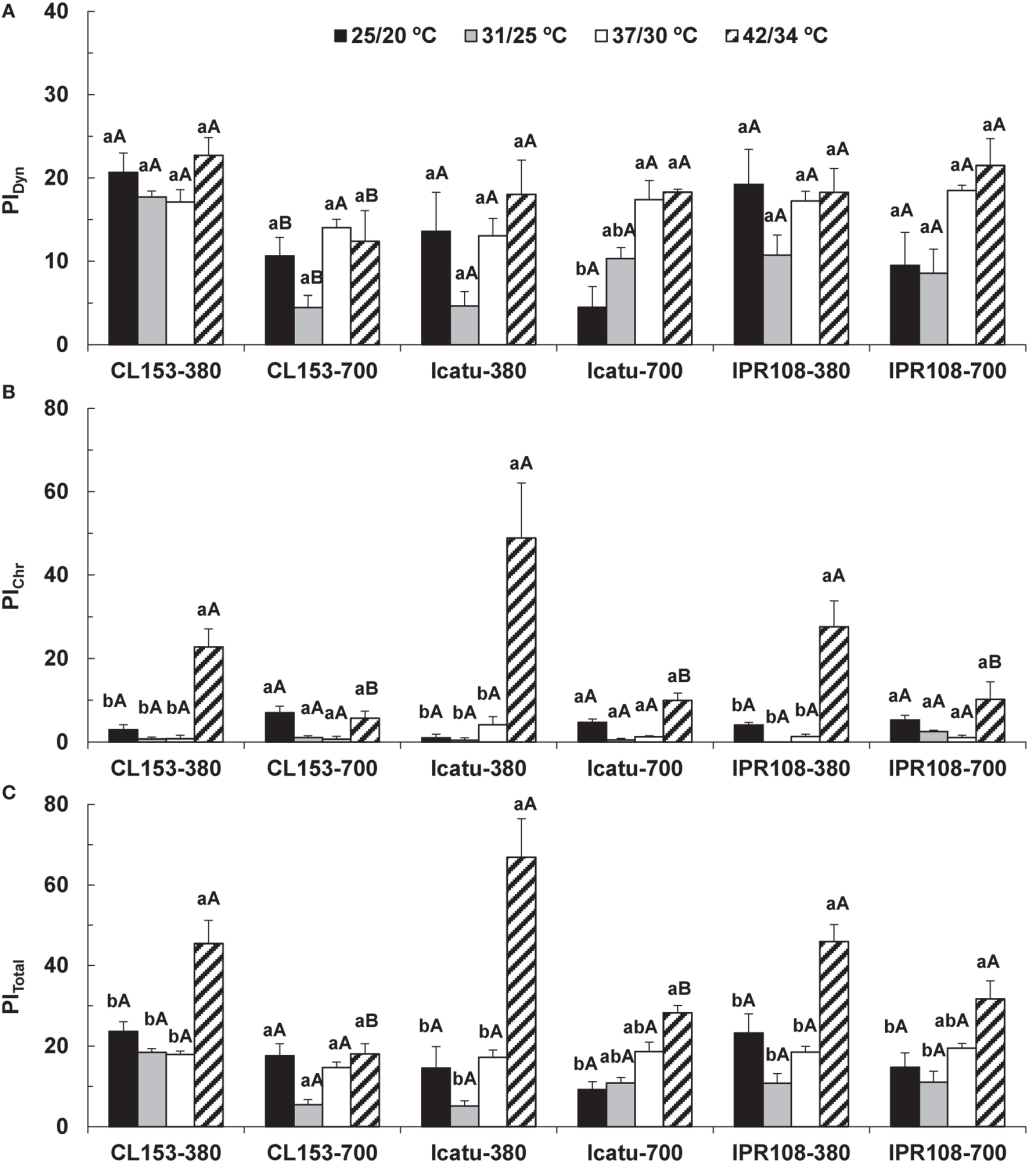
**Leaf fluorescence parameters related to the photoinhibition status of PSII in ***C. canephora*** cv. Conilon (CL153) and ***C. arabica*** (Icatu and IPR108) plants, which were grown under 380 or 700 μL CO_**2**_ L^**−1**^ and submitted to control (25/20°C, day/night) and supra-optimal temperatures of 31/25°C, 37/30°C, or 42/34°C**. The parameters include the calculation of (**A**) PI_Dyn_, dynamic photoinhibition, (**B**) PI_Chr_, chronic photoinhibition, and (**C**) PI_Total_, total photoinhibition. For each parameter, the mean values ± SE (*n* = 5–8) followed by different letters express significant differences between temperature treatments for the same CO_2_ level, separately for each genotype (a, b), or between CO_2_ levels for each temperature treatment, separately for each genotype (A, B).

Overall, dynamic PSII photoinhibition (PI_Dyn_) was mostly unresponsive to temperature and [CO_2_] (Figure 1A). Even so, CL153 380-plants maintained higher PI_Dyn_ values than those of under 700 μL CO_2_ L^−1^ throughout the experiment. In contrast, chronic PSII photoinhibition (PI_Chr_) was manifested almost exclusively at 42/34°C (Figure 1B) and in the 380-plants, particularly in Icatu. Reflecting PI_Dyn_ and PI_Chr_ variations at 42/34°C, the total photoinhibition (PI_Total_) showed significantly higher values in the 380-plants of all genotypes (maximal in Icatu), as well as in the 700-plants of *C. arabica* genotypes (Figure 1C).

### Photosynthetic pigment

At control temperature (25/20°C), enhanced [CO_2_] promoted a consistent tendency toward higher contents of several carotenoids (Car) in CL153 (Table 2), although significantly only for neoxanthin and carotenes. No changes were observed in total Chl contents (Table 3). The *C. arabica* plants showed a somewhat different pattern by the maintenance or even slight lower Car contents under high [CO_2_], namely in the pool of xanthophyll cycle pigments (VAZ), carotenes, and total Car.

**Table 2:** **Changes in leaf content (mg g^**−1**^ DW) of xanthophylls and carotenes, obtained through HPLC determinations in ***C. canephora*** cv. Conilon (CL153) and ***C. arabica*** (Icatu and IPR108) plants, grown under 380 or 700 μLCO_**2**_ L^**−1**^, at control (25/20°C, day/night) and supra-optimal temperatures of 31/25°C, 37/30°C, and 42/34°C**.

**Pigment**	**Genotype**	**[CO_2_] (μL L^−1^)**	**Temperature (day/night)**							
			**25/20°C**		**31/25°C**		**37/30°C**		**42/34°C**	
Neoxanthin (mg g^−1^ DW)	CL 153	380	0.206 ± 0.019	aB	0.225 ± 0.019	aA	0.255 ± 0.021	aA	0.245 ± 0.019	aA
		700	0.267 ± 0.019	aA	0.216 ± 0.021	abA	0.233 ± 0.021	abA	0.182 ± 0.019	bB
	Icatu	380	0.216 ± 0.012	aA	0.224 ± 0.010	aA	0.245 ± 0.010	aA	0.241 ± 0.010	aA
		700	0.218 ± 0.012	bA	0.223 ± 0.010	bA	0.263 ± 0.010	aA	0.252 ± 0.010	abA
	IPR 108	380	0.276 ± 0.016	aA	0.311 ± 0.016	aA	0.322 ± 0.016	aA	0.276 ± 0.014	aA
		700	0.216 ± 0.014	bB	0.291 ± 0.016	aA	0.266 ± 0.014	abB	0.239 ± 0.014	abA
Zeaxanthin (mg g^−1^ DW)	CL 153	380	0.041 ± 0.007	bA	0.046 ± 0.009	bA	0.036 ± 0.007	bA	0.085 ± 0.009	aA
		700	0.057 ± 0.007	aA	0.030 ± 0.007	aA	0.042 ± 0.009	aA	0.039 ± 0.009	aB
	Icatu	380	0.053 ± 0.009	abA	0.037 ± 0.009	bA	0.032 ± 0.010	bB	0.083 ± 0.010	aA
		700	0.066 ± 0.010	abA	0.039 ± 0.009	bA	0.064 ± 0.010	abA	0.075 ± 0.009	aA
	IPR 108	380	0.032 ± 0.010	bA	0.028 ± 0.008	bA	0.101 ± 0.009	aA	0.080 ± 0.008	aA
		700	0.040 ± 0.009	bA	0.040 ± 0.008	bA	0.041 ± 0.008	bB	0.089 ± 0.008	aA
V + A + Z (mg g^−1^ DW)	CL 153	380	0.318 ± 0.022	aA	0.318 ± 0.022	aA	0.342 ± 0.026	aA	0.288 ± 0.022	aA
		700	0.341 ± 0.026	aA	0.249 ± 0.026	abB	0.288 ± 0.026	abA	0.221 ± 0.022	bB
	Icatu	380	0.375 ± 0.019	abA	0.409 ± 0.019	aA	0.337 ± 0.019	bB	0.306 ± 0.019	bA
		700	0.300 ± 0.022	bB	0.407 ± 0.019	aA	0.416 ± 0.022	aA	0.312 ± 0.019	bA
	IPR 108	380	0.451 ± 0.031	abA	0.482 ± 0.031	aA	0.494 ± 0.027	aA	0.348 ± 0.027	bA
		700	0.338 ± 0.027	aB	0.428 ± 0.031	aA	0.392 ± 0.027	aB	0.349 ± 0.031	aA
DEPS	CL 153	380	0.169 ± 0.026	bA	0.198 ± 0.030	bA	0.186 ± 0.026	bA	0.359 ± 0.030	aA
		700	0.214 ± 0.026	aA	0.118 ± 0.026	aB	0.224 ± 0.030	aA	0.204 ± 0.026	aB
	Icatu	380	0.180 ± 0.028	bA	0.103 ± 0.026	bA	0.121 ± 0.030	bA	0.337 ± 0.030	aA
		700	0.248 ± 0.030	abA	0.125 ± 0.026	cA	0.178 ± 0.030	bcA	0.307 ± 0.026	aA
	IPR 108	380	0.084 ± 0.024	cB	0.072 ± 0.019	cB	0.219 ± 0.022	bA	0.341 ± 0.019	aA
		700	0.187 ± 0.022	bA	0.136 ± 0.020	bA	0.124 ± 0.020	bB	0.337 ± 0.019	aA
Lutein (mg g^−1^ DW)	CL 153	380	0.622 ± 0.041	bA	0.709 ± 0.041	bA	0.756 ± 0.047	abA	0.886 ± 0.041	aA
		700	0.719 ± 0.047	aA	0.519 ± 0.041	bB	0.674 ± 0.047	abA	0.603 ± 0.041	abB
	Icatu	380	0.637 ± 0.037	cA	0.704 ± 0.037	cA	0.878 ± 0.037	bA	1.17 ± 0.037	aA
		700	0.560 ± 0.043	cA	0.690 ± 0.037	cA	0.911 ± 0.037	bA	1.15 ± 0.037	aA
	IPR 108	380	0.828 ± 0.061	bA	0.846 ± 0.053	bA	1.226 ± 0.053	aA	1.21 ± 0.053	aA
		700	0.672 ± 0.053	cA	0.846 ± 0.061	bcA	0.936 ± 0.053	abB	1.11 ± 0.053	aA
α-Carotene (mg g^−1^ DW)	CL 153	380	0.163 ± 0.032	bB	0.194 ± 0.032	abA	0.298 ± 0.037	aA	0.198 ± 0.032	abA
		700	0.271 ± 0.032	aA	0.190 ± 0.032	aA	0.273 ± 0.037	aA	0.179 ± 0.032	aA
	Icatu	380	0.177 ± 0.016	bA	0.164 ± 0.016	bA	0.310 ± 0.019	aA	0.145 ± 0.016	bA
		700	0.164 ± 0.019	bA	0.168 ± 0.016	bA	0.249 ± 0.016	aB	0.172 ± 0.019	bA
	IPR 108	380	0.243 ± 0.024	abA	0.264 ± 0.024	aA	0.256 ± 0.024	aA	0.160 ± 0.021	bA
		700	0.137 ± 0.021	bB	0.241 ± 0.024	aA	0.261 ± 0.024	aA	0.147 ± 0.021	bA
β-Carotene (mg g^−1^ DW)	CL 153	380	0.237 ± 0.015	aB	0.287 ± 0.015	aA	0.258 ± 0.018	aA	0.263 ± 0.015	aA
		700	0.286 ± 0.015	aA	0.226 ± 0.015	bB	0.234 ± 0.018	abA	0.195 ± 0.015	bB
	Icatu	380	0.240 ± 0.018	bA	0.294 ± 0.018	abA	0.315 ± 0.018	aA	0.330 ± 0.018	aA
		700	0.227 ± 0.018	bA	0.276 ± 0.018	abA	0.310 ± 0.018	aA	0.328 ± 0.018	aA
	IPR 108	380	0.330 ± 0.014	abA	0.341 ± 0.014	aA	0.368 ± 0.014	aA	0.285 ± 0.012	bA
		700	0.227 ± 0.012	bB	0.313 ± 0.014	aA	0.323 ± 0.012	aB	0.298 ± 0.012	aA
(α + β) Carotene (mg g^−1^ DW)	CL 153	380	0.400 ± 0.022	bB	0.481 ± 0.034	abA	0.556 ± 0.019	aA	0.461 ± 0.043	abA
		700	0.557 ± 0.043	aA	0.416 ± 0.037	abA	0.507 ± 0.023	aA	0.374 ± 0.041	bA
	Icatu	380	0.418 ± 0.025	bA	0.458 ± 0.013	bA	0.581 ± 0.030	aA	0.475 ± 0.036	abA
		700	0.371 ± 0.019	bA	0.444 ± 0.030	abA	0.559 ± 0.045	aA	0.484 ± 0.012	abA
	IPR 108	380	0.572 ± 0.036	aA	0.605 ± 0.027	aA	0.624 ± 0.016	aA	0.446 ± 0.023	bA
		700	0.364 ± 0.016	bB	0.553 ± 0.038	aA	0.550 ± 0.029	aA	0.445 ± 0.025	abA
(α/β) Carotene (g g^−1^)	CL 153	380	0.697 ± 0.089	bA	0.669 ± 0.089	bA	1.164 ± 0.102	aA	0.737 ± 0.089	bA
		700	0.882 ± 0.089	aA	0.821 ± 0.089	aA	1.169 ± 0.102	aA	0.879 ± 0.089	aA
	Icatu	380	0.742 ± 0.073	bA	0.570 ± 0.073	bcA	1.051 ± 0.084	aA	0.432 ± 0.073	cA
		700	0.639 ± 0.073	abA	0.612 ± 0.073	abA	0.855 ± 0.073	aA	0.523 ± 0.084	bA
	IPR 108	380	0.731 ± 0.077	aA	0.778 ± 0.077	aA	0.705 ± 0.077	aA	0.583 ± 0.067	aA
		700	0.499 ± 0.077	bB	0.763 ± 0.077	abA	0.818 ± 0.077	aA	0.495 ± 0.067	bA
Total carotenoids (mg g^−1^ DW)	CL 153	380	1.55 ± 0.12	aA	1.73 ± 0.12	aA	1.91 ± 0.13	aA	1.88 ± 0.12	aA
		700	1.75 ± 0.13	aA	1.43 ± 0.12	aA	1.70 ± 0.13	aA	1.38 ± 0.12	aB
	Icatu	380	1.63 ± 0.07	cA	1.79 ± 0.07	bcA	2.04 ± 0.07	abA	2.19 ± 0.07	aA
		700	1.39 ± 0.08	cB	1.89 ± 0.08	bA	2.24 ± 0.08	aA	2.20 ± 0.07	aA
	IPR 108	380	2.13 ± 0.14	bA	2.29 ± 0.14	bA	2.82 ± 0.12	aA	2.36 ± 0.14	abA
		700	1.59 ± 0.12	bB	2.12 ± 0.14	aA	2.14 ± 0.12	aB	2.14 ± 0.12	aA
(V + A + Z)/Total carotenoids (g g^−1^)	CL 153	380	0.205 ± 0.005	aA	0.184 ± 0.005	bB	0.179 ± 0.006	bA	0.153 ± 0.005	cA
		700	0.193 ± 0.006	aA	0.201 ± 0.006	aA	0.169 ± 0.006	bA	0.159 ± 0.005	bA
	Icatu	380	0.230 ± 0.006	aA	0.227 ± 0.006	aA	0.165 ± 0.006	bB	0.140 ± 0.006	cA
		700	0.216 ± 0.007	aA	0.232 ± 0.006	aA	0.186 ± 0.007	bA	0.143 ± 0.006	cA
	IPR 108	380	0.213 ± 0.006	aA	0.210 ± 0.006	aA	0.173 ± 0.005	bA	0.149 ± 0.006	cA
		700	0.212 ± 0.005	aA	0.203 ± 0.005	aA	0.182 ± 0.005	bA	0.163 ± 0.005	bA

*For each parameter, the mean values ± SE (n = 6) followed by different letters express significant differences between temperatures for the same CO_2_ treatment, separately for each genotype (a, b, c, d), or between CO_2_ treatments for each temperature, separately for each genotype (A, B)*.

**Table 3:** **Changes in leaf content of total chlorophyll, Chl (***a*** + ***b***) (mg g^**−1**^ DW), and in the ratios of ***a***-to-***b*** chlorophyll and of total chlorophyll-to-total carotenoids, obtained through spectrophotometric determinations, in ***C. canephora*** cv. Conilon (CL153) and ***C. arabica*** (Icatu and IPR108) plants, grown under 380 or 700 μLCO_**2**_ L^**−1**^, at control (25/20°C, day/night) and supra-optimal temperatures of 31/25°C, 37/30°C, and 42/34°C**.

**Pigment**	**Genotype**	**[CO_2_] (μL L^−1^)**	**Temperature (day/night)**							
			**25/20°C**		**31/25°C**		**37/30°C**		**42/34°C**	
Chl (*a* + *b*) (mg g^−1^ DW)	CL 153	380	10.39 ± 0.70	aA	11.41 ± 0.81	aA	11.76 ± 0.81	aA	11.87 ± 0.70	aA
		700	10.38 ± 0.81	aA	9.40 ± 0.70	aA	10.94 ± 0.81	aA	8.30 ± 0.70	aB
	Icatu	380	9.81 ± 0.51	bA	11.00 ± 0.51	abA	12.16 ± 0.51	aA	11.06 ± 0.51	abA
		700	9.08 ± 0.51	bA	10.73 ± 0.51	abA	11.56 ± 0.59	aA	11.81 ± 0.51	aA
	IPR 108	380	11.28 ± 0.64	cA	14.32 ± 0.74	abA	15.26 ± 0.74	aA	12.33 ± 0.74	bcA
		700	9.45 ± 0.64	bB	9.44 ± 0.64	bB	12.71 ± 0.64	aB	11.11 ± 0.64	abA
Chl (*a*/*b*) (g g^−1^)	CL 153	380	3.33 ± 0.04	aA	3.44 ± 0.04	aA	2.84 ± 0.04	bB	2.62 ± 0.04	cA
		700	3.40 ± 0.04	aA	3.28 ± 0.04	aB	3.07 ± 0.05	bA	2.70 ± 0.04	cA
	Icatu	380	3.29 ± 0.06	aA	3.20 ± 0.06	aA	3.22 ± 0.07	aA	2.85 ± 0.06	bA
		700	3.13 ± 0.06	aA	3.15 ± 0.06	aA	2.89 ± 0.06	bB	2.80 ± 0.06	bA
	IPR 108	380	3.41 ± 0.04	aA	3.29 ± 0.05	aA	2.98 ± 0.04	bA	2.67 ± 0.04	cA
		700	3.24 ± 0.04	aB	3.09 ± 0.04	abB	3.02 ± 0.04	bA	2.76 ± 0.04	cA
Chl (*a* + *b*)/total carotenoids (g g^−1^)	CL 153	380	4.98 ± 0.09	aA	5.08 ± 0.10	aA	5.10 ± 0.10	aA	4.80 ± 0.09	aA
		700	4.89 ± 0.09	aA	5.11 ± 0.09	aA	5.10 ± 0.09	aA	4.90 ± 0.10	aA
	Icatu	380	4.92 ± 0.10	aA	4.97 ± 0.10	aA	4.83 ± 0.10	aA	4.25 ± 0.10	bA
		700	4.78 ± 0.10	aA	4.78 ± 0.10	aA	4.73 ± 0.12	aA	4.23 ± 0.10	bA
	IPR 108	380	4.82 ± 0.12	aA	5.07 ± 0.14	aA	4.79 ± 0.14	abA	4.35 ± 0.12	bA
		700	4.78 ± 0.12	aA	5.21 ± 0.12	aA	4.91 ± 0.12	aA	4.17 ± 0.12	bA

*For each parameter, the mean values ± SE (n = 6) followed by different letters express significant differences between temperatures for the same CO_2_ treatment, separately for each genotype (a, b, c, d), or between CO_2_ treatments for each temperature, separately for each genotype (A, B)*.

In *C. canephora* plants exposed to supra-optimal temperatures, decreases in most pigments (neoxanthin, VAZ pool, lutein, β-, and total carotenes) were observed in the 700-plants. Given that the 380-plants maintained or slightly increased (lutein, carotenes) their pigment contents (when compared to 25/20°C), differences between CO_2_ conditions emerged for most Car, significantly for neoxanthin, VAZ pool, lutein, and total Car under 42/34°C.

In *C. arabica* genotypes, [CO_2_] treatment caused marginal/occasional changes in pigment composition at high temperatures. Such changes were mostly restricted to 700-plants of IPR108, which at 37/30°C showed lower contents of neoxanthin, VAZ pool, DEPS, lutein, β- and total carotenes, total Car, and Chl (*a* + *b*) relative to their 380-counterparts. However, no significant differences associated with [CO_2_] treatments could be found at 42/34°C. In contrast to [CO_2_] treatment, increasing temperature remarkably impacted the *C. arabica* genotypes regardless of [CO_2_], as denoted by the increases in total Chls and total Car, reaching maximal values at 37/30°C or 42/34°C. The rise in total Car was related to increases in neoxanthin, Z and, especially, lutein and β-carotene (Table 2). In fact, at 42/34°C, rises in lutein pools reached 84 and 105% in Icatu, and 47 and 65% in IPR108, for the 380- and 700-plants, respectively, whereas the β-carotene pools increased ca. 40% in Icatu under both [CO_2_] conditions. In IPR108, maximal increases in β-carotene were found at 37/30°C, although with significance only for the 700-plants (42%).

Specifically regarding the photoprotective Z, moderately low values were maintained over the course of the experiment, but at 42/34°C significant Z increases (in parallel with moderate increases in DEPS) were observed in Icatu and IPR108 regardless of [CO_2_], as well as in CL153 380-plants. Furthermore, at 42/34°C most pigments (with the referred exceptions related to lutein and β-carotene) tended to lower concentrations than those observed at 37/30°C.

A moderate rise in total Chl was observed in *C. arabica* genotypes for both CO_2_ treatments, mostly until 37/30°C, as compared to 25/20°C values. In CL153 temperature rising did not provoke significantl changes in total Chl content within each [CO_2_]. However, at 42/34°C the CL153 700-plants displayed lower Chl content than 380-plants, resulting from slight divergent shifts promoted by temperature in these plant groups (Table 3). The values of Chl (*a*/*b*) ratio were similar between [CO_2_], but decreased at the two highest temperatures in all genotypes. Finally, the total Chl-to-total Car ratio, reflecting the light capture-to-dissipation pigment ratio, was quite stable until 37/30°C, but strongly decreased at 42/34°C in the *C. arabica* genotypes. No differences were found in this ratio in response to [CO_2_] over the entire experiment in all genotypes.

### Antioxidant enzyme activities

The activities of the antioxidant enzymes (Table 4) showed some differences at control temperature among genotypes in response to [CO_2_], although the values were maintained within the range usually observed for genotypes of both *C. arabica* and *C. canephora* (Ramalho et al., 1998; Chaves et al., 2008; Fortunato et al., 2010; Pompelli et al., 2010). For all enzymes (Cu,Zn-SOD, APX, GR, CAT), IPR108 700-plants showed lower activities than 380-plants. In Icatu and CL153 that same trend was observed for GR and CAT, contrasting to higher activities observed in the 700-plants for APX and Cu,Zn-SOD (significantly only for the latter).

**Table 4:** **Changes in chloroplastic maximal activities of the enzyme antioxidants Cu,Zn-superoxide dismutase (Cu,Zn-SOD), ascorbate peroxidase (APX), glutathione reductase (GR), as well as in cellular catalase in ***C. canephora*** cv. Conilon (CL153) and ***C. arabica*** (Icatu and IPR108) plants, grown under 380 or 700 μLCO_**2**_ L^**−1**^, at control (25/20°C, day/night) and maximal temperature exposure (42/34°C)**.

**Enzyme**	**Genotype**	**[CO_2_] (μL L^−1^)**	**Temperature (day/night)**					
			**25/20°C**		**37/30°C**		**42/34°C**	
Cu,Zn-SOD (Units g^−1^ DW)	CL 153	380	449 ± 4	aB	423 ± 5	aA	426 ± 10	aB
		700	537 ± 7	aA	444 ± 7	bA	525 ± 8	aA
	Icatu	380	523 ± 4	bB	431 ± 14	cA	602 ± 9	aA
		700	648 ± 11	aA	413 ± 3	cA	498 ± 9	bB
	IPR 108	380	568 ± 14	aA	437 ± 9	bB	555 ± 13	aA
		700	450 ± 4	cB	694 ± 3	aA	577 ± 7	bA
APX (mmol ASC min^−1^ g^−1^ DW)	CL 153	380	7.23 ± 1.15	bA	13.05 ± 1.27	aA	3.94 ± 1.10	bA
		700	9.85 ± 0.61	aA	8.40 ± 0.78	aB	3.38 ± 0.29	bA
	Icatu	380	11.04 ± 1.66	aA	10.22 ± 0.96	aA	1.32 ± 0.22	bA
		700	14.88 ± 0.89	aA	10.97 ± 1.70	bA	1.07 ± 0.28	cA
	IPR 108	380	5.28 ± 0.76	aA	0.62 ± 0.21	bA	4.23 ± 0.38	aA
		700	1.68 ± 0.23	aB	1.92 ± 0.28	aA	2.18 ± 0.12	aB
GR (μmol NADPH min^−1^ g^−1^ DW)	CL 153	380	0.790 ± 0.110	bA	1.282 ± 0.108	aA	0.794 ± 0.088	bA
		700	0.435 ± 0.056	aA	0.571 ± 0.020	aB	0.455 ± 0.054	aA
	Icatu	380	1.179 ± 0.019	bA	1.816 ± 0.221	aA	0.395 ± 0.053	cB
		700	0.777 ± 0.084	bB	1.019 ± 0.118	abB	1.105 ± 0.037	aA
	IPR 108	380	1.396 ± 0.121	aA	0.696 ± 0.112	bA	0.440 ± 0.037	bA
		700	0.400 ± 0.019	aB	0.478 ± 0.032	aA	0.560 ± 0.028	aA
Catalase (μmol H_2_O_2_ min^−1^ g^−1^ DW)	CL 153	380	8.55 ± 0.93	bA	12.42 ± 0.85	aA	13.07 ± 0.96	aA
		700	4.67 ± 1.00	bB	10.49 ± 1.30	aA	13.48 ± 1.44	aA
	Icatu	380	7.62 ± 1.47	bA	17.72 ± 3.69	aA	5.37 ± 1.27	bB
		700	5.67 ± 1.19	bA	10.34 ± 0.88	aB	12.77 ± 0.54	aA
	IPR 108	380	17.87 ± 1.15	aA	14.88 ± 0.93	aB	13.57 ± 1.16	aB
		700	13.09 ± 0.69	bB	21.02 ± 2.33	aA	23.80 ± 0.43	aA

*For each parameter, the mean values ± SE (n = 4) followed by different letters express significant differences between temperatures for the same CO_2_ treatment, separately for each genotype (a, b), or between CO_2_ treatments for each temperature, separately for each genotype (A, B)*.

With temperature rise to 37/30°C, enzyme activities changed differently with respect to genotype and [CO_2_] conditions (Table 4). In CL153 380-plants, increases were found in APX, GR and CAT, whereas in the 700-plants only CAT activity was increased. In Icatu, increases in GR and CAT were observed under both CO_2_ conditions, whereas some decrease was found in Cu,Zn-SOD and APX (the latter only in the 700-plants). In IPR108, different patterns were observed related to [CO_2_], with activity increases in 700-plants (significantly for Cu,Zn-SOD and CAT), and decreases in 380-plants (non-significantly for CAT) in all enzymes.

Pronounced changes in enzyme activities were further noted when temperature increased from 37/30 to 42/34°C. In CL153 plants, regardless of [CO_2_], Cu,Zn-SOD and GR activities remained invariant in parallel with an enhanced CAT activity (53% for 380 plants and 189% for 700-plants), whereas APX activity was remarkably reduced (46 and 66% in 380- and 700-plants, respectively). Within *C. arabica* genotypes, Icatu displayed reductions in APX (near to 10% of the initial value, irrespective of [CO_2_]), GR and CAT (380-plants) and Cu,Zn-SOD (700-plants), in parallel with increases in both GR and CAT (700-plants) as well as in Cu,Zn-SOD (380-plants). In IPR108, the 700-plants showed increased activities of Cu,Zn-SOD (28%), APX (30%), GR (40%), and CAT, but only the activity of CAT varied significantly in response to [CO_2_] treatments.

In summary, APX was the most negatively affected enzyme at the highest temperature (in CL153 and Icatu), CAT activity increased or remained invariant, whereas Cu,Zn-SOD was the less responsive enzyme to heat stress.

### Other protective molecules

Ascorbate (ASC) content was similar between CO_2_ conditions within each genotype under control temperature. However, *C. arabica* genotypes showed ca. three-fold higher ASC contents than those in CL153 (Table 5). Overall, ASC pools were clearly reduced with increasing temperatures, beginning at 31/25°C for CL153 and IPR108 700-plants, and for Icatu 380-plants. Remarkable decreases were observed at the two highest temperatures, ranging from 69 (IPR108-700) to 86% (CL153-380), as compared with the control temperature.

**Table 5:** **Changes in the cellular content of the non-enzyme antioxidant molecules ascorbate and α-tocopherol, as well as HSP70 and the soluble sugars stachyose and, raffinose in ***C. canephora*** cv. Conilon (CL153) and ***C. arabica*** (Icatu and IPR108) plants, grown under 380 or 700 μLCO_**2**_ L^**−1**^, at control (25/20°C, day/night) and supra-optimal temperatures of 31/25°C, 37/30°C, and 42/34°C**.

**Compound**	**Genotype**	**[CO_2_] (μL L^−1^)**	**Temperature (day/night)**							
			**25/20°C**		**31/25°C**		**37/30°C**		**42/34°C**	
Ascorbate (mg g^−1^ DW)	CL 153	380	0.316 ± 0.042	aA	0.341 ± 0.037	aA	0.070 ± 0.034	bA	0.044 ± 0.039	bA
		700	0.406 ± 0.037	aA	0.158 ± 0.037	bB	0.064 ± 0.042	bA	0.069 ± 0.042	bA
	Icatu	380	0.994 ± 0.117	aA	0.430 ± 0.104	bB	0.660 ± 0.117	abA	0.248 ± 0.135	bA
		700	1.136 ± 0.135	aA	0.900 ± 0.104	abA	0.453 ± 0.135	bcA	0.224 ± 0.125	cA
	IPR 108	380	1.288 ± 0.089	aA	1.248 ± 0.089	aA	0.800 ± 0.079	bA	0.347 ± 0.103	cA
		700	1.205 ± 0.103	aA	0.545 ± 0.089	bcB	0.761 ± 0.103	bA	0.374 ± 0.103	cA
α-Tocopherol (mg g^−1^ DW)	CL 153	380	0.729 ± 0.035	aA	0.659 ± 0.043	abA	0.403 ± 0.043	cA	0.565 ± 0.043	bcA
		700	0.414 ± 0.043	bcB	0.583 ± 0.043	aA	0.271 ± 0.043	cB	0.503 ± 0.043	abA
	Icatu	380	0.284 ± 0.036	cA	0.341 ± 0.036	bcA	0.458 ± 0.036	bA	1.013 ± 0.036	aA
		700	0.379 ± 0.036	bcA	0.338 ± 0.036	cA	0.489 ± 0.029	bA	0.716 ± 0.036	aB
	IPR 108	380	0.373 ± 0.017	cA	0.494 ± 0.017	bA	0.473 ± 0.017	bA	0.813 ± 0.017	aA
		700	0.206 ± 0.017	dB	0.337 ± 0.017	cB	0.502 ± 0.017	bA	0.578 ± 0.017	aB
HSP70 (μg g^−1^ DW)	CL 153	380	0.667 ± 0.087	cB	2.051 ± 0.019	aA	1.771 ± 0.028	bA	0.812 ± 0.014	cA
		700	0.994 ± 0.035	bA	1.533 ± 0.040	aB	1.609 ± 0.046	aA	0.861 ± 0.011	bA
	Icatu	380	0.818 ± 0.047	bA	1.810 ± 0.041	aA	1.699 ± 0.047	aB	1.897 ± 0.060	aA
		700	0.710 ± 0.040	cA	1.771 ± 0.049	bA	3.264 ± 0.084	aA	1.774 ± 0.056	bA
	IPR 108	380	1.001 ± 0.017	cA	1.635 ± 0.060	bB	1.868 ± 0.012	aA	1.616 ± 0.036	bB
		700	0.830 ± 0.047	cA	1.877 ± 0.021	aA	1.558 ± 0.038	bB	1.960 ± 0.048	aA
Stachyose (mg g^−1^ DW)	CL 153	380	1.85 ± 0.06	aA	2.05 ± 0.09	aA	1.41 ± 0.02	bA	0.65 ± 0.06	cB
		700	0.79 ± 0.06	dB	1.74 ± 0.09	aB	1.43 ± 0.02	bA	1.13 ± 0.06	cA
	Icatu	380	1.04 ± 0.07	bA	0.44 ± 0.01	cA	1.09 ± 0.05	abA	1.22 ± 0.04	aA
		700	0.39 ± 0.01	cB	0.32 ± 0.01	cA	0.75 ± 0.03	bB	1.34 ± 0.03	aA
	IPR 108	380	1.17 ± 0.02	cA	1.37 ± 0.04	bcA	1.52 ± 0.03	bA	2.04 ± 0.20	aA
		700	0.61 ± 0.04	bB	0.67 ± 0.04	bB	1.11 ± 0.01	aB	0.98 ± 0.02	abB
Raffinose (mg g^−1^ DW)	CL 153	380	3.54 ± 0.12	abA	2.14 ± 0.20	cA	2.64 ± 0.36	bcB	4.53 ± 0.21	aA
		700	3.65 ± 0.12	abA	2.71 ± 0.20	bA	3.93 ± 0.36	aA	4.69 ± 0.21	aA
	Icatu	380	4.06 ± 0.13	bA	4.54 ± 0.44	bA	10.71 ± 0.09	aA	11.07 ± 0.22	aB
		700	3.62 ± 0.18	dA	5.02 ± 0.44	cA	8.84 ± 0.29	bB	13.14 ± 0.15	aA
	IPR 108	380	4.98 ± 0.08	dA	7.32 ± 0.35	cA	11.20 ± 0.28	bA	15.18 ± 0.73	aA
		700	5.11 ± 0.10	cA	5.66 ± 0.35	cB	7.49 ± 0.14	bB	12.94 ± 0.18	aB

*For each parameter, the mean values ± SE (n = 4–6) followed by different letters express significant differences between temperatures for the same CO_2_ treatment, separately for each genotype (a, b, c, d), or between CO_2_ treatments for each temperature, separately for each genotype (A, B)*.

α-tocopherol (TOC) showed different trends between the two coffee species. Despite some fluctuations with rising temperatures, at 42/34°C TOC pools changed moderately in CL153 irrespective of [CO_2_], with a 22% decrease in 380-plants and a 21% rise in 700-plants. In contrast, at 42/34°C and under both CO_2_ conditions, TOC pools increased in Icatu and IPR108, ranging from 89 (Icatu-700) to 257% (Icatu-380), with 380-plants presenting the highest contents.

At 25/20°C the 700-plants showed similar (raffinose) or lower (stachyose) contents of RFOs than their 380-plant counterparts. Temperature rise prompted modifications in RFOs, with no clear relation with the [CO_2_] treatments. In CL153, significant increases were observed only for stachyose in the 700-plants from 31/25°C onwards. On the other hand, at 42/34°C, both stachyose and raffinose displayed greater contents in *C. arabica* plants, when compared to 25/20°C, particularly raffinose whose contents increased between 153% (IPR108-700) and 263% (Icatu-700). Also, in IPR108, both RFOs content were always higher in the 380-plants at supra-optimal temperatures.

The constitutive cell HSP70 pools (25/20°C) were similar between [CO_2_] in *C. arabica* genotypes, but presented a higher value in CL153 700-plants. Rising temperature clearly promoted HSP70 accumulation: significantly higher HSP70 contents were already observed at 31/25°C in all genotypes, which were mostly maintained until 42/34°C (*C. arabica* plants) or until 37/30°C in CL153 (decreasing afterwards). In all genotypes, maximal HSP70 contents, relative to control temperature, were observed in CL153-380 at 31/25°C (208%), and in the 700-plants of Icatu at 37/30°C (360%) and IPR108 at 42/34°C (136%).

### Expression of genes with a potential role in heat acclimation

Several changes in gene expression patterns were imposed by enhanced [CO_2_] and temperature (Table 6). At 25/20°C, the expression of genes coding for the protective proteins *HSP70, ELIP, Chape 20* and *Chape 60* was similar or lower in the 700-plants compared with their 380-plant counterparts in all genotypes. With rising temperature a global gene upregulation was observed (maximal at 42/34°C) in all genotypes and both [CO_2_], although stronger in the 700-plants of Icatu and CL153 and 380-plants of IPR. Notably, CL153 700-plants showed the highest increases: ca. 33- (*Chape 60*), 55- (*ELIP*), 59- (*HSP70*), and 145- (*Chape 20*) fold.

**Table 6:** **Real-time PCR expression studies relative to the expression value observed under control conditions of temperature and CO_2_ (25/20°C, 380 μL CO_2_ L^−1^), within each genotype from leaves of *C. canephora* cv. Conilon (CL153) and *C. arabica* (Icatu and IPR108) plants, grown under 380 or 700 μLCO_2_ L^−1^, at control (25/20°C, day/night) and supra-optimal temperatures of 31/25°C, 37/30°C, and 42/34°C**.

**Genotype**	**Temperature (day/night)**	**[CO_2_] (μL L^−1^)**	**Gene expression relative to control temperature and CO_2_ (25/20°C, 380 μL L^−1^)**											
			***HSP70***	***ELIP***	***Chape 20***	***Chape 60***	***CAT***	***CuSOD1***	***CuSOD2***	***FeSOD***	***APX Cyt***	***APX Chl***	***TOC Mt***	***TOC Cyt***
CL 153	25/20°C	380	1.00	1.00	1.00	1.00	1.00	1.00	1.00	1.00	1.00	1.00	1.00	1.00
		700	0.53	0.68	1.28	0.24	0.85	0.16	0.49	1.64	7.53	1.17	0.84	0.17
	31/25°C	380	0.60	1.82	3.56^*^	0.52	1.70	0.02^*^	0.61	1.14	3.26	1.18	0.06^*^	0.35
		700	0.64	1.35	0.63	0.37	1.36	0.15	0.56	0.59	2.71	1.07	0.29	0.18
	37/30 °C	380	1.93	3.33	7.48^*^	1.14	1.73	0.02^*^	1.10	0.94	39.96^*^	1.35	0.09	0.24
		700	2.37	6.77	3.76	1.53	1.64	0.16	1.10	1.29	81.88^*^	3.21	0.95	0.36
	42/34°C	380	7.39	8.30	16.64^*^	6.24	2.37^*^	0.08	3.32	1.28	251.02^*^	4.44	0.39	0.43
		700	59.04^*^	54.57^*^	145.31^*^	33.14^*^	10.02^*^	0.59	24.26^*^	5.18	1761.06^*^	9.59^*^	2.67	2.30
Icatu	25/20°C	380	1.00	1.00	1.00	1.00	1.00	1.00	1.00	1.00	1.00	1.00	1.00	1.00
		700	0.63	1.67	0.81	0.75	2.05^*^	0.46	0.80	0.75	0.85	1.36	0.87	0.78
	31/25°C	380	0.95	2.33	1.93	1.18	1.88^*^	0.29^*^	1.00	0.39^*^	0.46^*^	0.72	0.46^*^	0.98
		700	0.87	2.70	1.74	1.32	1.88^*^	0.19^*^	0.95	0.48^*^	1.15	0.91	0.47	1.12
	37/30 °C	380	2.70^*^	4.71^*^	4.00^*^	2.83^*^	1.84^*^	0.19^*^	1.39^*^	0.59^*^	0.50	0.71	0.44	0.74
		700	2.74	7.38	3.29^*^	3.42	2.86^*^	0.13^*^	2.13^*^	0.77	1.57	2.87	0.22^*^	1.65
	42/34°C	380	4.96^*^	9.28^*^	7.40^*^	14.63^*^	1.75^*^	0.07^*^	2.88^*^	0.47^*^	7.94^*^	1.48	1.31	1.40
		700	9.36^*^	12.16^*^	13.52^*^	34.19^*^	1.28	0.15^*^	3.75^*^	0.49	24.49^*^	5.04^*^	0.62	1.57
IPR 108	25/20°C	380	1.00	1.00	1.00	1.00	1.00	1.00	1.00	1.00	1.00	1.00	1.00	1.00
		700	0.37^*^	1.41	0.36^*^	0.27^*^	1.66^*^	0.19^*^	0.58^*^	0.44^*^	0.27^*^	0.23^*^	0.49	0.28^*^
	31/25°C	380	0.40	1.62^*^	1.07	0.78	1.55	0.57	0.94	0.47^*^	0.90	0.93	0.59	0.85
		700	0.50^*^	1.24	0.82	1.36	2.89^*^	0.66	1.17	0.54^*^	0.42^*^	0.23^*^	0.25^*^	0.33^*^
	37/30 °C	380	2.03^*^	6.51^*^	4.18^*^	3.08^*^	2.03^*^	4.13^*^	0.79	0.44^*^	0.55	1.26	0.89	0.77
		700	1.07	3.13	1.30	1.86	0.97	0.57	1.12	0.30^*^	0.14^*^	0.35	0.21^*^	0.25^*^
	42/34°C	380	4.53^*^	11.23^*^	9.85^*^	20.83^*^	1.67	0.06	1.98^*^	0.49^*^	17.37^*^	1.40	0.66	1.00
		700	1.79	4.18	4.12^*^	4.25	0.64	0.96	0.85	0.26	2.73	0.61	0.03^*^	0.24^*^

*It were studied genes of the 70 kDa heat shock-related protein from chloroplastic stroma (HSP70), early light-induced protein (ELIP), 20 kDa chaperonin from chloroplast (Chape 20), Chaperonin CPN60 (Chape 60), all related to protective proteins; the genes of catalase isozyme 1 (CAT), Cu,Zn superoxide dismutases (CuSOD1 and CuSOD2), Fe-superoxide dismutase from chloroplast (FeSOD), ascorbate peroxidase from cytoplasm (APX Cyt) and chloroplast (APX Chl), all related to antioxidative enzymes; and probable tocopherol O-methyltransferase from chloroplast (Toc Mt) and tocopherol cyclase from chloroplast (Toc Cy) related to the tocopherol synthesis pathway. The gene expression values represent n fold relative to the control of temperature and [CO_2_] (25/20°C–380 μL CO_2_ L^−1^) within each genotype. Original expression values for each gene resulted from the mean ± SE (n = 6–9), from 3 independent biological assays*.

* *Indicate the presence of statistical significance*.

Overall, the expression patterns of genes related to antioxidative system components (*CAT, SOD, APX*, and *TOC*) tended to be lower in the 700-plants than in the 380-plants at the control temperature, mainly in the two *C. arabica* genotypes (except for *CAT*). Under rising temperatures *CAT* expression increased moderately (below three-fold) in most cases, and more markedly in CL153 700-plants (10-fold). The *APX* transcripts (particularly *APX Cyt*) increased with heat, with maximal abundance at 42/34°C, especially in Icatu and CL153 700-plants. In fact, the *APX Cyt* upregulation in CL153-700 (1760-fold) was, by far, the largest observed over the entire experiment for all studied genes. In contrast, *CuSOD1* and *FeSOD* genes were mainly downregulated by temperature in all genotypes. Among the genes coding for SOD enzymes, only *CuSOD2* was significantly upregulated at the two highest temperatures in both [CO_2_] in Icatu, and at 42/34°C in CL153-700 and IPR-380 plants. *TOC* genes were mainly downregulated (except CL153 700-plants). Notably, IPR108 had the strongest global downregulation for the genes related to the antioxidative system, with the exception of *APX Cyt* at 42/34°C in 380-plants.

## Discussion

### PSII photoinhibition in face of high CO_2_ and temperature

The balance of energy use and/or dissipation capabilities, herein analyzed using the dynamic (PI_Dyn_) and chronic (PI_Chr_) PSII photoinhibition estimates (Figures 1A,B), remained nearly invariant until 37/30°C, a temperature that is considered well above the optimum for both *C. arabica* and *C. canephora* (DaMatta and Ramalho, 2006). These results evidence a considerable heat tolerance of the coffee's photosynthetic machinery until 37/30°C, which is accompanied by the maintenance of photochemical energy use (Rodrigues et al., 2016). In fact, photochemical components and RuBisCO activity were strengthened (especially under high [CO_2_]), in contrast to what is often observed in other species even at moderately high temperature (e.g., Haldimann and Feller, 2004). In sharp contrast, increases in both PI_Chr_ (and PI_Total_) were evident at 42/34°C (Figures 1B,C) in 380-plants, particularly in Icatu. This reflected a mitigatory impact of elevated [CO_2_] on the photochemical functioning, in line with the lower rate constant for PSII inactivation (F_s_/${\text{F}}_{\text{m}}^{'}$) and non-photochemical quenching attributable to photo-inactivation and non-regulated energy dissipation in PSII (Y_(NO)_) observed in 700 plants at 42/34°C (Rodrigues et al., 2016). Taking all of this information together, it is clear that tolerance to heat stress was compromised at 42/34°C at normal [CO_2_], whereas at elevated [CO_2_] a mitigating effect against heat impacts was evident in this study. Similar findings have been reported in other species, where elevated [CO_2_] has been shown to preserve the photosynthetic functioning, as in the Mediterranean cork oak (*Quercus suber*) under the exposure to supra-optimal temperatures (Faria et al., 1996).

### Triggering heat-protective mechanisms under enhanced [CO_2_]

Thermotolerance frequently involves the triggering of cellular mechanisms that prevent oxidative damage, interlinking temperature stress, and a balance between ROS signaling and control (Suzuki and Mittler, 2006; Hasanuzzaman et al., 2013). In addition, the strengthening of antioxidative mechanisms has proven to be decisive to stress acclimation in *Coffea* spp., namely, to cold, high irradiance, drought and nitrogen starvation (Ramalho et al., 1998, 2014; Fortunato et al., 2010; Batista-Santos et al., 2011; Cavatte et al., 2012). Therefore, under enhanced [CO_2_], robust thermal protective mechanisms are expected, supporting an unaltered photosynthetic functioning up to 37/30°C in addition to maintaining a better metabolic performance even at 42/34°C, when compared to normal [CO_2_], as reported previously (Rodrigues et al., 2016).

#### Pigment dynamics

Carotenoids act as thermal quenchers and scavengers of highly reactive species of Chl and O_2_. Under control temperature, the high [CO_2_] prompted a tendency to higher carotenoid contents in CL153. This contrasted with a reduction trend in photoprotective carotenoids (VAZ pool, lutein, carotenes) in *C. arabica* genotypes, similarly to what has been found in other species, where a relaxation of the antioxidant system was associated with a higher C-assimilation that ultimately led to a reduced energy load on the photosynthetic apparatus (Erice et al., 2007).

Under supra-optimal temperatures, decreases of most carotenoids were found in the 700-plants of CL153 (and IPR108 until 37/30°C), when compared to their respective 380-plants. In contrast, *C. arabica* genotypes showed increased total Chl and total Car contents regardless of [CO_2_], suggesting a photosynthetic structures strengthening at 37/30°C (and even at 42/34°C in some cases) driven by temperature. Increases in total Car resulted mostly from the moderate Z increases and the strong rises in lutein and β-carotene that have complementary actions, decreasing the formation of highly reactive species of Chl and oxygen, preventing PSs and membrane photodamage, and protecting the Cyt b_6_/f complex from photobleaching promoted by ^1^O_2_ (Zhang et al., 1999). Z protects LHCs from both PSs by controlling ^3^Chl^*^ and ^1^Chl^*^ formation and by removing epoxy groups from the oxidized double bonds from the lipid phase of thylakoid fatty acids, whereas β-carotene and lutein-epoxide cycle (and neoxanthin) quenches ^3^Chl^*^ and ^1^O_2_, which are lipoperoxidation initiators (Lichtenthaler and Babani, 2004; Logan, 2005; Kalituho et al., 2007; Matsubara et al., 2011). In addition, neoxanthin maintenance and lutein increase might improve antennae functionality given that these xanthophylls are integral components of the peripheral LHC and are important for its trimeric assembly, stability, and efficiency (Kalituho et al., 2007; Matsubara et al., 2011). These functions agree with the maintenance of PSI and PSII activities in *C. arabica* genotypes until 37/30°C (Rodrigues et al., 2016). Nevertheless, at 42/34°C PSII photoinhibition was evident in 380-plants (Figure 1), when pigments (except lutein) tended to lower values (Table 2) when compared with those observed at 37/30°C. Therefore, changes in photoprotective pigments were not strong enough to protect the PSs in the three genotypes, especially in Icatu, which was the most affected genotype by heat stress. However, the lower photoinhibition status in the 700-plants at 42/34°C (Figure 1) was not accompanied by clear increases in the pools of photoprotective pigments, suggesting that these molecules are not the only ones accounting for the better preservation of photosynthetic activity under heat stress at elevated [CO_2_].

Chl-to-Car ratio was quite stable along the experiment in CL153, and until 37/30°C in *C. arabica* plants (Table 3). This pattern was independent from [CO_2_], as also observed in other woody species (Bader et al., 2010). Chl (*a*/*b*) ratio showed similar values between [CO_2_], but decreased in all genotypes at the two highest temperatures, especially at 42/34°C. Since net degradation of Chls was apparently absent, this Chl (*a*/*b*) reduction reflected preferential Chl *b* synthesis, suggesting functional readjustments with a higher proportion of light harvesting chlorophyll-protein complex from PSII (LHCII), which contains the majority of Chl *b* (with a Chl *a*/*b* ratio around 1.1–1.3) (Lichtenthaler and Babani, 2004). Nonetheless, it should not be ruled out that decreases in Chl (*a*/*b*) might reflect some heat susceptibility given that this ratio has been shown to increase in heat-tolerant genotypes of other species (Bita and Gerats, 2013).

#### Antioxidative enzymes

The ascorbate-glutathione cycle is a vital mechanism to control cell oxidative stress, and include both enzyme (e.g., Cu,Zn-SOD, APX, GR, among others) and non-enzyme (ASC, GSH) components, which altogether scavenge several ROS (${\text{O}}_{2}^{\cdot –}$, H_2_O_2_, OH^·^) (Asada, 1994; Foyer, 2002; Smirnoff, 2005). With the exception of Cu,Zn-SOD in CL153 and Icatu, 700-plants showed a somewhat downregulation of the antioxidative enzymatic system at control temperature. Such lower constitutive level of antioxidative capability under high [CO_2_] has been interpreted in other species as compromising the plant's ability to cope with sudden stress events (Pritchard et al., 2000). However, in coffee plants, this may be associated with higher photosynthetic rates (Rodrigues et al., 2016), which concomitantly with the inhibition of photorespiration under elevated CO_2_ (DaMatta et al., 2016), is believed to decrease the excitation pressure in the PSs and avoid ROS formation, thus ultimately precluding the need of developing a robust antioxidant system. In fact, although elevated CO_2_ may lead to decreases in SOD, APX, GR, and CAT activities (Pritchard et al., 2000; Erice et al., 2007; Vurro et al., 2009), even under supra-optimal temperatures (Erice et al., 2007; AbdElgawad et al., 2015), a concomitant reduction in the lipoperoxidation status has been often observed. Taken together, this information supports the notion that reduced ROS production under high CO_2_ conditions in C_3_ plants is indeed related to a higher photosynthetic functioning and inhibition of photorespiration (Erice et al., 2007; Vurro et al., 2009; AbdElgawad et al., 2015), as would also be the case in coffee plants at control temperature.

With rising temperatures thermotolerance can be improved by increasing transcript and protein levels of ROS-scavenging enzymes (Suzuki and Mittler, 2006; Hasanuzzaman et al., 2013), which agrees with the increases in APX, GR, and CAT in CL153-380, GR and CAT in Icatu (both CO_2_ conditions), and Cu,Zn-SOD and CAT in IPR108 700-plants. In addition, in line with the observed heat tolerance until 37/30°C the 380-plants from Icatu presented similar (Cu,Zn-SOD and APX) or higher (GR and CAT) activities than their 700-plants counterparts, compensating for the lower photosynthetic rates. Similarly, the IPR108 380-plants presented higher (Cu,Zn-SOD and CAT) or similar (GR and APX) enzyme activities than their respective 700-plants. Irrespective of genotypes and [CO_2_], FeSOD gene was not significantly upregulated under supra-optimal temperatures, and therefore ${\text{O}}_{2}^{\cdot –}$ removal through extra chloroplast SOD action is not expected to be reinforced.

The additional temperature increase to 42/34°C led to further changes in enzyme activities with varying genotypic patterns. In CL153 plants only APX activity was reduced (despite the *APX Chl* upregulation), but the H_2_O_2_ control might have been achieved in extra-chloroplast compartments in good agreement with both increases in CAT activity in both [CO_2_] and upregulation of *CAT* and *APX Cyt* genes (especially in the 700-plants) (Table 6). This responsiveness to H_2_O_2_ control, together with the stability in Cu,Zn-SOD and GR, could have alleviated heat stress in these plants by maintaining redox homeostasis (Li et al., 2014) and minimizing the heat-induced oxidative impairments to the photosynthetic apparatus (Figure 1; Rodrigues et al., 2016). These responses are in line with the well-known better heat tolerance of *C. canephora* (DaMatta and Ramalho, 2006). On the other hand, Icatu 380-plants showed reductions in the activity of all enzymes (except Cu,Zn-SOD), likely contributing to the strongest decrease in photosynthetic performance (Rodrigues et al., 2016) and highest photoinhibition status (Figure 1) relative to the other genotypes. Furthermore, in Icatu 380-plants the increase in Cu,Zn-SOD, but not in APX and CAT activities, might have limited an integrated H_2_O_2_ scavenging (Fortunato et al., 2010). Compared with Icatu 380-plants, their 700-counterparts (despite showing decreased APX activity) displayed a strong upregulation of GR and CAT activities, as well as *APX Cyt* (and *APX Chl*) gene expression at 42/34°C, which may have contributed to a lower impact on the photosynthetic performance in elevated [CO_2_]. At the two highest temperatures, the scavenging capability in IPR108 700-plants was not stronger than that of 380-plants (with the exception related to CAT activity), and a lower gene expression for the antioxidant enzymes and other protective proteins (HSP70, ELIP, Chape 20, and 60) was observed. Therefore, a better ROS control could be mostly prompted by the higher rates of electrons driven to C-assimilation, as also reported in grapevine (*Vitis vinifera*) under high [CO_2_] and increased temperature (Salazar-Parra et al., 2012).

Chloroplastic APX was the most affected enzyme at 42/34°C, contrasting to the large increase in *APX Cyt* expression, which was the most responsive gene to high temperature (in CL153). Since CAT was the only enzyme to present significant increased activities in the three genotypes in the 700-plants (and also in CL153 380-plants), this suggested a drift of H_2_O_2_ control from chloroplast APX to extra-chloroplast CAT (predominantly located in peroxisomes, glyoxissomes, and mitochondria) and possibly cytosolic APX. In fact, since H_2_O_2_ is capable of diffusing passively across membranes, extrachloroplastic scavenging systems are important H_2_O_2_ detoxification pathways (Feierabend, 2005; Logan, 2005).

#### Other protective molecules

ROS control can be complemented by ASC, TOC, HSPs, and other cellular protectants under stressful conditions (Suzuki and Mittler, 2006). The changes found in other protective molecules were mostly promoted by temperature rather than by enhanced [CO_2_] *per se*. This was the case of ASC, which decreased significantly with supra-optimal temperatures in all genotypes, which could have been promoted by TOC accumulation (Kanwischer et al., 2005) in *C. arabica* plants. At the highest temperature, ASC reductions were in line with losses in APX activity, as observed in CL153 and Icatu. Similar results have also been reported in other species (Erice et al., 2007). In fact, ASC reacts with H_2_O_2_ in a reaction catalyzed by APX, as well as non-enzymatically with ^1^O_2_, O_2_i^−^, OHi, and lipid hydroperoxides (Asada, 1994; Foyer, 2002; Logan, 2005). Given that ASC is also a substrate for V de-epoxidase to synthesize Z from V (Logan 2005; Smirnoff 2005), decreases in ASC pools might also have limited Z synthesis, thus ultimately resulting in a modest DEPS value (Table 2) under heat stress.

In plants, TOC pools are confined to chloroplasts (Munné-Bosch, 2005). Therefore, the remarkable increase of this lipophilic antioxidant under heat stress in *C. arabica*, especially in the 380-plants at 42/24°C, clearly reflected a positive stress response at chloroplast level, given that TOC deactivates ^1^O_2_, ${\text{O}}_{2}^{•–}$, OH^•^, limits lipid peroxidation by reducing lipid peroxyl radicals, and stabilizes membrane structures due to its interactions with polyunsaturated fatty acyl chains (Munné-Bosch, 2005; Smirnoff, 2005). This action was further reinforced by the presence of high β-carotene (Table 2) in *C. arabica* genotypes (mostly in Icatu) that co-operate in limiting ^1^O_2_ damages (Munné-Bosch, 2005) and regenerating carotenoid radicals produced with the reaction of carotenoid with lipid peroxyl radicals (Smirnoff, 2005). This TOC increase contrasted with an absence of upregulation of *TOC My* and *TOC Cyt* genes (genes coding for enzymes that catalyse the last two steps of TOC synthesis) in *C. arabica* plants regardless of [CO_2_]. However, this apparent discrepancy may be related to the observation that these final steps might be not limiting to TOC synthesis (Kanwischer et al., 2005).

The RFOs pathway is highly adjustable in plant stress response. The only RFOs detected in coffee leaves, stachyose, and raffinose, increased at the two highest temperatures. RFOs typically accumulate under environmental stresses (Sicher, 2013), having membrane protective roles (including the maintenance of thylakoid electron transport) against temperature, drought, and ROS impairments (Santarius, 1973; Santos et al., 2011; Sicher, 2013). Therefore, their increases were consistent with the preservation of thylakoid electron transport involving both photosystems capabilities in coffee (Rodrigues et al., 2016). Nevertheless, in IPR108, despite the buildup in stachyose and raffinose contents promoted by high temperature, it seems noteworthy that high [CO_2_] plants maintained significantly lower values than 380-plants. Assuming that these leaf metabolites accumulation was related to activation of stress related genes, a lower buildup under heat stress conditions could point that CO_2_ enrichment was able to mitigate this process, as suggested to occur in soybean leaflets, where high temperature promoted raffinose increase under ambient CO_2_, but not under high [CO_2_] (Sicher, 2013).

Plants evolved also molecular chaperones as stress defenses. These are a group of functional proteins with key roles in protein protection in both optimal and adverse conditions (Wang et al., 2004), including HSPs that are related to thermal acclimation. HSP70 prevents proteins of different metabolic pathways from denaturation and aggregation, helping in folding, refolding, and assembly, as well as in translocation processes and in facilitating the proteolytic degradation of unstable proteins (Wang et al., 2004; Fragkostefanakis et al., 2015). In coffee plants HSP70 content was not constitutively buildup by high [CO_2_], similar to the findings of Bokhari et al. (2007) in rice, accompanied by a downregulation tendency of HSP70 gene at 25/20°C in the 700-plants relative to the 380-ones. However, HSP70 synthesis was one of the earlier responses to rising temperature, from 31/25 upwards. Although some *HSP70* downregulation occurred under high CO_2_ at control temperature, increased transcriptional levels were found at the two highest temperatures for all genotypes. Furthermore, although this response was not clearly related to high CO_2_,higher HSP70 gene upregulation were observed in 700-plants of CL153 and Icatu at 42/34°C. Similar strong and fast transcriptional activation has been considered essential for protein homeostasis and a requisite for development and survival under stress (Fragkostefanakis et al., 2015). HSP70 can also be involved in the protection and repair of PSII (Schroda et al., 1999) in addition to having a pivotal role in the upregulation of enzymatic antioxidant defenses, under single or combined drought and heat stresses, thereby indirectly helping ROS control (Hu et al., 2010). Therefore, HSP70 increases might have contributed to preserve PSII functioning at supra-optimal temperatures (Figure 1; Rodrigues et al., 2016) until 37/30°C for both [CO_2_], and to stimulate GR and CAT activities at 42/34°C (700-plants) (Table 4).

Concerning other protective molecules, the gene expression of chaperonins 20 and 60 (*Chape 20* and *60*) and early light-induced protein (*ELIP*) from the chloroplast was also examined (Table 6). As for HSP70, while significant changes in *Chape 20* and *60* showed a clear upregulation under elevated CO_2_ at 42/34°C (in CL153 and Icatu), under control temperature the expression did not significantly change, or even tend to decrease, with CO_2_ enrichment, as also observed by Vicente et al. (2015) in durum wheat. In this study, a 4°C increase further downregulated *Chape 60* transcription, especially under low N-availability. However, both chaperonin 20 and 60 have been associated with enhancement of stress tolerance as they play a crucial role by assisting a wide range of newly synthesized and newly translocated proteins to achieve their native forms, namely RuBisCO (see Wang et al., 2004). Furthermore, chaperonin 60 is also involved in the assembly of chloroplast ATP synthase (Mao et al., 2015). Therefore, the strong upregulation *Chape 20* and *Chape 60* at the two highest temperatures, especially in 700-plants of CL153 and Icatu, could helped to maintain ATP synthesis coupled to thylakoid electron transport, a lower PSII inactivation and higher photosynthetic potential, as observed at 42/24°C in these plants (Rodrigues et al., 2016).

The ELIP family members are nuclear-encoded photoprotective proteins that accumulate in thylakoid membranes in response to various abiotic stresses. They prevent free radical formation and participate in energy dissipation (Adamska, 2001). Indeed a strong relation between tolerance to photooxidation and ELIPs level has been reported (Hutin et al., 2003). Thus, the stronger *ELIP* upregulation on the 700-plants of CL153 and Icatu might have played a role in preserving thylakoid functions (Rodrigues et al., 2016), thus avoiding photoinhibition (Figure 1).

## Conclusions

Relevant heat tolerance up to 37/30°C for both [CO_2_] and all coffee genotypes was observed, largely supported by the maintenance or increase of the pools of several protective molecules (neoxanthin, lutein, carotenes, TOC, HSP70, raffinose), by the activities of antioxidant enzymes (SOD, APX, QR, CAT), and by the upregulated expression of some genes (ELIP, Chape 20). Nevertheless, *C. arabica* plants seemed to be more responsive, although improved photosynthetic activity was promoted under high [CO_2_] in all genotypes (Rodrigues et al., 2016). However, at 42/34°C photosynthesis photoinhibition was manifested, especially in the 380-plants and in Icatu. At this temperature a global reinforcement of the antioxidative system was not observed, but gene upregulation of protective mechanisms (*HSP70*, chaperonins, *ELIP*, and *APX Cyt*), as well as raffinose content, constituted a common heat defense line in all genotypes. A consistently higher expression was observed in CL153 at the highest temperature, in agreement with its better tolerance to elevated temperatures. TOC and HSP70 seemed to be particularly relevant in *C. arabica* genotypes. The presence of RFOs, lutein, β-carotene, TOC, and HSP70, as well as the upregulated expression of genes related to protective proteins (*ELIPS, HSP70, Chape 20*, and *60*) and antioxidant enzymes (*CAT, CuSOD2, APX Cyt, APX Chl*), which should act in concert to control ROS formation and scavenging, were mostly driven by temperature increase. Nevertheless, it was noteworthy that the plants grown under enhanced [CO_2_] maintained higher activities of GR (Icatu), CAT (Icatu and IPR), and kept (or even increased) the activities of Cu,Zn-SOD, APX, and CAT. These differences between [CO_2_] were particularly clear in Icatu, with the 380-plants suffering from the greatest photosynthetic impact amongst the genotypes. Furthermore, the strongest upregulation of genes related to protective proteins found in the 700-plants of CL153 and Icatu likely contributed to strengthen their ability to maintain higher functional levels. The simultaneous gene upregulation of antioxidative enzymes and molecular chaperones at the two highest temperatures fully agrees with the known pivotal protective roles of these proteins, as well as to the observed cross-talk between HSPs/chaperones and other stress response mechanisms under abiotic stress conditions (Wang et al., 2004).

Notably, our data also suggest a drift of H_2_O_2_ control from chloroplast (APX) to extra-chloroplast (CAT) taking into account the increases in CAT activities in all 700-plants (and CL153 380-plants) coupled with strong decreases in APX activity. The 700-plants showed higher photochemical energy use ability, which should constitute the major sink for electron transport, preventing electrons to react with O_2_ to produce ROS. Therefore, this should be perceived as a first line of defense against excessive energy load, constituting a primary mean of protection against oxidative conditions and photoinhibition, allowing acclimation to take place.

Overall, our results showed that high [CO_2_] mitigated the heat impact through higher photosynthetic functioning, upregulation of protective molecules, as well as through higher activity of some antioxidant enzymes. Such high [CO_2_]-dependent effects on antioxidant defenses likely favored the maintenance of ROS at controlled levels and would in turn justify the lower heat impact on the photosynthetic components under elevated [CO_2_] (Rodrigues et al., 2016). Therefore, our findings clearly extend our understanding why high [CO_2_] constitutes a key player to coffee heat resilience and acclimation, what is clearly relevant in the context of predicted future global warming scenarios for the coffee crop sustainability.

## Author contributions

According to their competences, all authors contributed transversally to the several stages of the work, including its design, data acquisition, analysis and interpretation, critically review of the manuscript, and approval of the submitted version. Furthermore, they agree to be accountable for all aspects of the work in ensuring that questions related to the accuracy or integrity of any part of the work were appropriately investigated and resolved.

## Funding

This work was supported by national funds from Fundação para a Ciência e a Tecnologia through the projects PTDC/AGR-PRO/3386/2012, the research units UID/AGR/04129/2013 (LEAF) and UID/GEO/04035/2013 (GeoBioTec), as well through the grant SFRH/BPD/47563/2008 (AF) co-financed through the POPH program subsidized by the European Social Fund. Brazilian funding from CAPES (grants PDSE: 000427/2014-04, W.P. Rodrigues; 0343/2014-05, MM; 12226/12-2, LM), CNPq and Fapemig (fellowships to FDM, FP, and EC) are also greatly acknowledged.

### Conflict of interest statement

The authors declare that the research was conducted in the absence of any commercial or financial relationships that could be construed as a potential conflict of interest.

## Abbreviations

A: antheraxanthin
APX: ascorbate peroxidase
ASC: ascorbate
Chl: chlorophyll
^3^Chl^*^: triplet state of Chl
Chl (a+b): Total Chl
Cu, Zn-SOD, Cu: Zn-superoxide dismutase
DHAR: dehydroascorbate reductase
GR: glutathione reductase
g_s_: stomatal conductance
H_2_O_2_: hydrogen peroxide
^1^O_2_: singlet oxygen
${\text{O}}_{2}^{•}$: superoxide anion radical
^•^OH: hydroxyl radical
PI_Chr_, PI_Dyn_, and PI_Tot_, chronic photoinhibition, dynamic photoinhibition: and total photoinhibition
SD: stomatal density
SI: stomatal index
ROS: reactive oxygen species
TOC: α-tocopherol
V: violaxanthin
Z: zeaxanthin.
